# Observations on Certain of the Diseases of Young Children

**Published:** 1850-12

**Authors:** 


ART. IV.—*Observations on certain of the Diseases of young Children.* By CHARLES D. MEIGS, M. D., Professor of the Diseases of Women and Children in the Jefferson Medical College at Philadelphia, &c. &c. Philadelphia: Lea & Blanchard. 1850.

These "observatious" are the result of an opportunity presented by an early commencement of the Lecture term in the Jefferson school, in 1849, for delivering a few preliminary discourses, before the usual lectures were commenced. We are glad that any occasion occurred to enable Professor Meigs to publish this book, for many useful and valuable practical indications may be found in these pages, by a man who has the art and patience to work his way through the language. It is strange and pitiable that a man who sees as clearly, and thinks as well as Professor Meigs does, should seek to clothe his thoughts in the livery which he makes them wear. For his own sake, and the advancement of medical science, we wish he would seek some form of the English language thnt approaches more nearly that in common use, than that of which he is so fond. Those readers, however, who may take the trouble to peruse this book, will find it replete with instruction. We do not wish to be understood as endorsing all the pathological opinions of Professor Meigs, that are scattered through these pages, but the practical remarks are generally sound, and may be safely followed. We have been especially pleased with the remarks on coryza, bowel complaints, jaundice, cyanosis neonati, respiratory disorders, whooping-cough, laryngismus and scarlatina, and these constitute the largest portion of the work.

We earnestly wish the Professor could impart to others those astonishing powers of diagnosis, which he claims to possess himself. He claims that there is a language in ear-ache, which reveals to his tutored ear, the nature of the disorder, before he gets inside the house. This manifests an extreme education of the ear, we mean of the Professor, which, unfortunately, but few have attained. We frankly confess we have met with difficulty in this particular diagnosis, even after we had got into the room of the sufferer, and in full hearing of most inordinate crying. We have not always been able to tell whether the discordant speech was from the belly or the ear. This kind of translation is very difficult at times, much more so indeed than Greek declensions. But inspired by the Professor's example we shall apply ourself diligently to study, and work up our aural apparatus to a comprehension of the most delicate shades of the varieties of a baby's crying. If we succeed, it will be an achievement worthy of Laennec's school itself.

We hope no reader of the observations of Professor Meigs, on scarlatina, will be frightened from his practical details, by his singularly wild pathology of the disease. Though that has no basis, the views of treatment have a solid and substantial foundation.

We take much pleasure in recommending this excellent little work to the attention of medical practitioners. It deserves their attention, and after they commence its perusal, they will not willingly abandon it, until they have mastered its contents. We read the work, while suffering severely from a carbuncle, and its fascinating pages often beguiled us into forgetfulness of agonizing pain. May it teach others to relieve the afflictions of the young.

